# Complete Nucleotide Sequence and Molecular Characterization of *Bacillus* Phage TP21 and its Relatedness to Other Phages with the Same Name

**DOI:** 10.3390/v2040961

**Published:** 2010-04-06

**Authors:** Jochen Klumpp, Richard Calendar, Martin J. Loessner

**Affiliations:** 1 Institute of Food, Nutrition and Health, ETH Zurich, 8092 Zurich, Switzerland; E-Mail: martin.loessner@ethz.ch; 2 University of California, 510 Barker Hall, Berkeley, CA 94720, USA; E-Mail: rishard@berkeley.edu

**Keywords:** TP21, *Bacillus*, bacteriophage

## Abstract

Three different *Bacillus* bacteriophages designated TP21 are known from the literature. We have determined the sequence and structure of the TP21-L genome, and compared it to the other phages. The genome is 37.5 kb in size, possesses fixed invariable genome ends and features the typical modular organization of a temperate siphovirus. TP21-L is neither identical to TP21 isolated by Thorne (TP21-T), as shown by a PCR-based approach nor to TP21 isolated by He *et al.* (TP21-H), as estimated from phage dimensions. For reasons of clarity, we suggest renaming the different TP21 isolates.

## 1. Introduction

*Bacillus anthracis*, *B. cereus* and *B. thuringiensis* are closely related organisms and represent a diverse range of pathogens as well as biotechnologically useful, non-pathogenic bacteria [1–4]. Bacteriophages and their components can be very useful tools for typing, detection and control of pathogenic bacteria in the food chain, biodefense measures, and for treatment of human infections. Thus, a better knowledge of the phages infecting *Bacillus* bacteria is highly desirable [5–8].

It is unfortunate that, over the last decades, the name TP21 has been assigned to three independently isolated phages infecting *B. anthracis*, *B. thuringiensis* and *B. cereus* [9–11]. However, it may be assumed that all of them are different, based on electron microscopy and/or partial genome sequences available [10]. The aim of this study was to determine the complete genome sequence of a specific TP21 phage isolate previously studied in our laboratory [10]. For reasons of clarity, we added the modifier “L” to the phage name, *i.e.*, naming it TP21-L.

## 2. Results and Discussion

**Figure 1.**
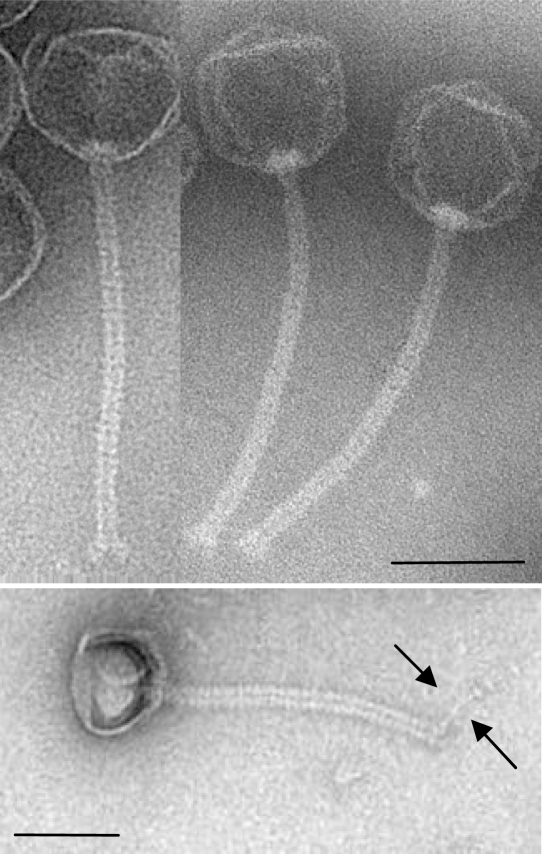
Transmission electron micrographs of TP21-L virions negatively stained with 2% uranyl acetate or 2% ammonium molybdate. The bar represents 50 nm. A putative tail fiber structure is indicated by arrows.

TP21-L was propagated using host HER1399 (ATCC 13472) in liquid culture [10], and purified by poly-ethylene glycol precipitation and density gradient centrifugation as described elsewhere [12]. Electron microscopy of purified negatively stained phage particles [13,14] (Figure 1), allowed assignment of TP21-L to the *Siphoviridae* family, in the order of the *Caudovirales* [15]. The phage features an isometric head of 58.5 nm diameter and a long, non-contractile, flexible tail of 144.8 nm length and 11.0 nm diameter. A putative tail fiber could be observed (Figure 1, indicated by arrows), corresponding to a putative tail fiber encoding gene in the genome of TP21-L (see below).

**Figure 2.**
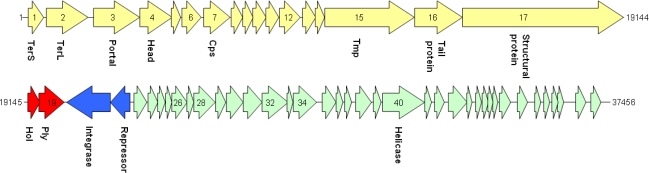
Genome map of TP21-L. The lysogeny control region is shown in blue, the lysis cassette in red. Structural genes (late genes) are depicted in yellow and putative early genes involved in DNA recombination, replication and modification in green. Abbreviations are: TerS: Terminase small subunit. TerL: Terminase large subunit. Cps: Major capsid protein. Tmp: Tail tape measure protein. Hol: Holin. Ply: Endolysin. Numbers to both sides of the graph indicate nt position in the genome sequence. Genes are numbered according to annotation.

**Figure 3.**
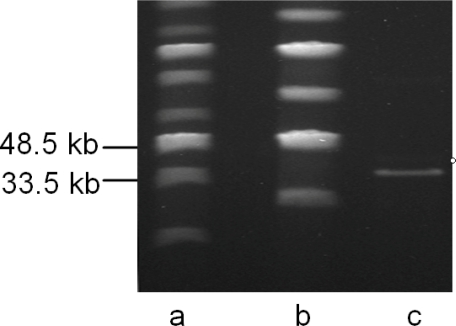
Pulsed-field gel electrophoresis of full length TP21-L genomic DNA prepared by phenolic extraction. **a.** and **b**. MidRange PFG Marker I and II (New England Biolabs, Switzerland). **c.** TP21-L DNA

Phage genomic DNA was extracted from purified phage as described elsewhere [12]. The TP21-L genome sequence was determined using a shotgun cloning and sequencing approach, followed by manual gap closing using primer walking. After assembly of the complete genome with 5.6-fold average coverage, TP21-L revealed a unit genome sequence of 37,456 base pairs (Figure 2), which is in good agreement with pulsed field gel electrophoresis analysis of full length phage DNA [13] (Figure 3). The G+C content is 37.8 mol%, slightly higher than that of the host bacteria (35.5 mol%, as determined from Genbank data of published host bacteria genomes (NC_003909, NC_004742, NC_005957, NC_003997, and NC_008600). The sequence of TP21-L has been deposited in the databases under accession number **EU887664**.

**Figure 4.**
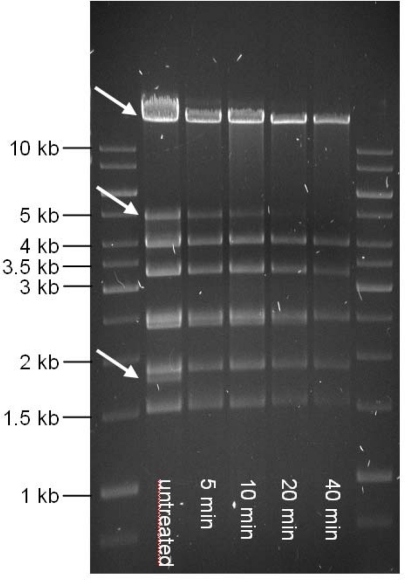
Gel electrophoresis of TP21-L DNA predigested with Bal31 nuclease (New England Biolabs), phenolized and subsequently digested with PvuI restriction endonuclease (Fermentas). Marker: 1kb Marker (Fermentas). Other lanes: untreated DNA and PvuI digest of DNA pretreated with Bal31 for indicated time. Arrows indicate fragments disappearing over time.

The TP21-L DNA molecule features invariable, fixed ends, as revealed by the typical pattern of single fragments disappearing over time when Bal31-predigested DNA is subjected to restriction digestion [13] (Figure 4). Heating of restriction digests prior to electrophoresis did not reveal any change compared to non-heated samples (data not shown), suggesting the absence of cohesive ends [16].

**Figure 5.**
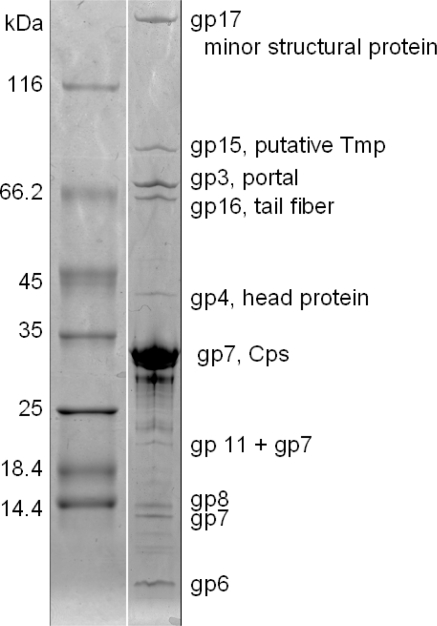
SDS-PAGE of TP21-L structural proteins. Left: Size Marker, molecular mass is indicated. Right: TP21-L structural proteins and their assignment as deduced from MALDI-MS/MS analysis. Abbreviations: Cps: Major capsid protein. Tmp: Tail tape measure protein.

Fifty-six putative open reading frames could be identified in the TP21-L genome, but only 17 of them revealed homologies to proteins of other bacteriophages (Table 1). No indication for modified, hydroxy-methylated bases [17] was found by restriction profiling. Mass spectrometry based identification (MALDI-MS peptide fingerprinting) of SDS-PAGE-separated structural proteins (Figure 5) [13,18] led to the identification of ten distinct protein species: one could be allocated to a minor structural protein (gp17) of 192.6 kDa, and another could be allocated to the putative tape measure protein (gp15) of 99.9 kDa. The dimension of the phage tail is in good correlation with the size of the Tmp protein, as demonstrated for phage Lambda and others [19–21] (Table 1, Figure 2). The putative portal protein (gp3) was identified in a band of approx 67 kDa, not in agreement with the predicted mass, and a tail fiber protein (Figures 1, 2) (gp16) was identified in a band of approx. 58 kDa. A putative capsid protein (gp4) as well as the major capsid protein (gp7) were also identified (39 kDa and 30 kDa, respectively). Yet another band (approximately 21 kDa) apparently contained traces of the major capsid protein, probably due to proteolytic cleavage or posttranslational modification of Cps, a fact also observed in other phages [13,22]. The predicted gene products gp6, gp8, and gp11 could also be identified as structural proteins, but do not exhibit amino acid homology to any known protein from the databases. However, the predicted mass of gp6 did not match the observed mass (Figure 5), suggesting posttranslational processing of this protein.

From the genome sequence (Figure 2), the endolysin (gp19) [10,23], a putative holin (gp18; featuring a TMHMM-predicted transmembrane domain [24]), as well as the lysogeny control region could be deduced. The latter consists of only two genes (gp20 and gp21) (Figure 2), whose predicted products share homologies with a transcription repressor and a recombinase. This correlates well with the ability of TP21-L to lysogenize its host (HER1399). In order to demonstrate this, we have isolated clones of HER1399 resistant to infection with TP21-L, sub-cultured the cells, treated them with UV light (254 nm, 120 mJ/cm^2^, 2 min), and used the sterile-filtered supernatants for demonstration of lytic activity against HER1399 on pre-inoculated agar plates. As a control, a UV-induced culture of wild-type HER1399 did not release any phage. The lysogenized strains were homoimmune to superinfection with up to 10^9^ Pfu/ml TP21-L. PCR using TP21-L specific primers (see below) confirmed the presence of phage in the lysogenized strains (results not shown).

Unfortunately, the designation TP21 has coincidentally been assigned to a total of three phages in the past. In our attempt to identify the individual TP21 isolates and clarify their nomenclature, the origins and history of the isolates is briefly described. TP21-L was made available to our lab by Hans-Wolfgang Ackermann, from the Félix d’Herelle Reference Center for Bacterial Viruses in 1994; it was described as a phage infecting *Bacillus thuringiensis* and designated TP21. Unfortunately, it was not possible to further elucidate the exact origin of this phage, and it was also not kept in the Félix d’Herelle Reference Center for Bacterial Viruses collection. However, since the genome sequence is now available, and it is also clear that TP21-L is unique among the *Bacillus* phages, it should be re-deposited in the collection.

**Figure 6.**
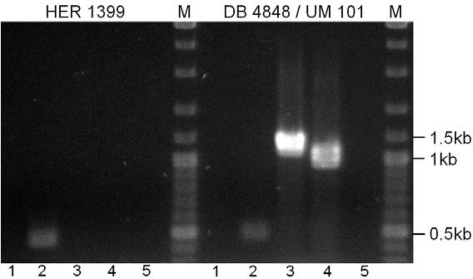
PCR detection of TP21-T. Left block: A colony of HER1399 was used as template. Right block: A colony of DB 4848 / UM 101 was used as template. M: 1kb Marker (Fermentas). 1. Primer combination TP21-L1_Fw and R; 2: Primer combination TP21-L2_Fw and R; 3: Primer combination TP21-T1_Fw and R; 4: Primer combination TP21-T2_Fw and R. 5: Negative control (no primer).

The second *Bacillus* phage named TP21 was isolated more than 20 years ago by Ruhfel and Thorne [11,25]. We propose that this phage should be renamed to TP21-T. The phage is unusual since it was shown to persist as an autonomously replicating plasmidal prophage in *B. thuringiensis* kurstaki [25]. In order to determine that TP21-L is different from TP21-T, we designed and used a set of PCR primers to amplify unique sequences of 0.5 to 1.1 kb from the genomes of both phages (the primer sequences were generated based on the unpublished genome sequence of TP21-T (R. Okinaka and P. Jackson, personal communication). Amplification with primers specific for TP21-L phage do not yield detectable products using template DNA from *B. thuringiensis kurstaki* HD1-9 or strain DP 4848 / UM 101, a carrier of TP21-T (Figure 6), although the primer combination TP21-L2_Fw an TP21-L2_R seems to produce an unspecific product both with HER1399 and DB 4848 as template (Figure 6, lane 2). Using primer combinations specific for TP21-T produced products of the expected size with strain DB 4848 as template but not with HER1399 (Figure 6). Vice versa, using TP21-L DNA as template did also not yield specific amplification products with TP21-T-specific primers [11] (data not shown). We conclude that TP21-L is not the same as TP21-T from Ruhfel and Thorne [11,25].

The third TP21 isolate was isolated from a Chinese factory producing *B. thuringiensis* powder and described by He and coworkers [9], and thus renamed to TP21-H. This phage apparently features an elongated head of 87 x 55 nm and a flexible tail of 140 x 8 nm in size (B2 morphotype), thus clearly distinguishing it from TP21-L (Figure 1). Unfortunately, however, further details are not available, and the whereabouts of TP21-H are unclear. It is not kept in the Félix d’Herelle Reference Center for Bacterial Viruses collection or any other collection, and has probably been lost (H.-W. Ackermann, personal communication). Even though, we propose to name this phage TP21-H, should it ever be rediscovered or reisolated.

## 3. Experimental Section

### 3.1. Phage Propagation

Phage TP21-L was propagated on *B. cereus* HER1399 (ATCC 13472) at 30°C in liquid culture in half-strength BHI media (Biolife, Italy) under light agitation. Phages were PEG precipitated (PEG 8000, Fluka, Switzerland) over night at 0°C from cleared lysates and purified by CsCl density gradient centrifugation [12] in a Beckmann L-60 ultracentrifuge at 76.000 x g for 18 h.

### 3.2. Transmission Electron Microscopy

Electron micrographs of purified TP21-L particles were taken as previously described [13,14]. TP21-L was negatively stained with 2% uranyl acetate or 2% ammonium molybdate. Samples were observed with a Philips CM100 microscope (FEI, USA) at 100 kV equipped with a TVIPS Fastscan charge-coupled-device camera (Tietz Systems, Germany).

### 3.3. DNA Isolation and Sequencing

TP21-L DNA was extracted from CsCl purified phage after dialysis against 2.000-fold excess of SM buffer (100 mM NaCl, 8 mM MgSO_4_, 50 mM Tris-Cl) at 4°C by organic extraction [12]. Briefly, following EDTA (0.5 M) and Proteinase K (50 μg/ml) treatment at 56°C for 1 h, DNA was extracted by subsequent steps of phenol, phenol-chloroform and chloroform-isoamyl alcohol (Roth, Germany) addition, centrifugation and removal of the organic phase, followed by ethanol precipitation [12]. For the preparation of phage DNA shotgun libraries, DNA was sonicated (Sonopuls, Bandelin, Germany) to 1–3 kb fragment size, size-exclusion selected by electrophoresis, blunt-ended (EndIt Repair Kit, Epicentre, USA) and ligated into EcoRV (Fermentas, Germany)-linearized pBLUESCRIPT II SK minus (Stratagene, USA) vector, followed by electroporation into *E. coli* XL1-blue MRF’ and blue-white screening. Confirmed inserts were sequenced using primers M13forward (GTAAAACGACGGCCAGT) and M13reverse (CAGGAAACAGCTATGACC). Gaps remaining between contigs were closed by a primer walking strategy using purified genomic phage DNA as template. Primers were derived from the contig sequences as they became available.

### 3.4. Peptide Mass Fingerprinting

Phage proteins were separated by horizontal sodium dodecyl sulfate-polyacrylamide gel electrophoresis on 8–18% gradient gels (GE Healthcare, Germany) as described previously [13]. Unstained Protein Molecular Weight Marker (Fermentas) was used as molecular size marker. Gels were stained with Coomassie blue R-350 (Phastblue R; GE Healthcare). Major bands were excised from the gel and, after tryptic in-gel digestion, analyzed by matrix-assisted laser desorption ionization-time of flight mass spectrometry (MALDI-MS/MS) to determine the peptide masses of the fragments (Functional Genomics Center Zurich, Switzerland). Data were analyzed using Scaffold 2 (Proteome Software Inc., USA) software. Protein domains were predicted with InterProScan (http://www.ebi.ac.uk/InterProScan).

### 3.5. Bal31 Nuclease Treatment, PCR and Electrophoresis

For determination of the genome structure of TP21-L, 20 μg of purified TP21-L DNA were treated with 10 U of Bal31 nuclease (Fermentas) at 30 °C. Samples were taken 0, 5, 10, 20 and 40 min after treatment and phenol-chloroform extracted [12], followed by subsequent restriction with PvuI (Fermentas) for 3 h at 37 °C and electrophoresis.

PCR was performed using 2x PCR Master Mix (Fermentas) according to the manufacturer’s instructions and 2 μl of one colony of bacterial strain dissolved in 100 μl water or approximately 1x10^7^ pfu phage were used as template. Primers used were TP21-L1_Fw (TCTGGTCAAGGTCGATATGG), TP21-L1_R (TGTATTTCCGTAGGTTTGCC), TP21-L2_Fw (CGGATGAAACGATCAAAGG) and TP21-L2_R (TGACTCACATTCCCACGG) for TP21-L and TP21-T1_Fw (GTACATACTGAT TTCACTGCTACC), TP21-T1_R (GGTAATTGGTCGTGTTGAGG), TP21-T2_Fw (GCTGTAT CAAATCCTAGAGAGC) and TP21-T2_R (AGCACACCTTATGAGTAGTAAGG) specific for TP21-T. We used an annealing temperature of 52°C and 1.5 min elongation time in a Biometra T3000 Cycler.

Pulsed-Field Gel Electrophoresis of purified phage DNA was performed in a CHEF-DR III apparatus (Biorad, Germany) at 1–5 s switch time, 120° angle and 5 V/cm in 14 °C 0.5x TBE buffer for 20 h. Conventional electrophoresis was done in a Pharmacia GNA-200 electrophoresis apparatus at 2 V/cm for 6 h in 1x TAE buffer.

### 3.6. Bioinformatics

Sequences were edited and aligned using the software Vector NTI Advance version 10.3 (Invitrogen, Switzerland) or CLC Main Workbench Version 5.5 (CLC Bio, Denmark). Open reading frames (ORFs) corresponding to a minimum size of 30 amino acids were predicted with Vector NTI or CLC Main Workbench using ATG, GTG, and TTG as possible start codons. The BLAST algorithms used for sequence homology searches are available through NCBI (http://www.ncbi.nlm.nih.gov), Vector NTI, or CLC Main Workbench.

## 4. Conclusions

We present the complete genome sequence and molecular characterization of the temperate *Bacillus* phage TP21-L. The TP21-L unit genome is 37.5 kb in size and encodes 56 open reading frames, 17 of which could be assigned a putative function. Ten structural proteins of the virion were identified by peptide mass fingerprinting. We analyzed the relationship of TP21-L to two *Bacillus* phages sharing the same designation. The three TP21 isolates analyzed here clearly represent different phages, as determined by genome sequencing of TP21-L, PCR probing of TP21-T and comparison of morphological data on TP21-H and TP21-L. Only two of them have been maintained, and they should be designated TP21-L and TP21-T. Unfortunately, the confusion in naming or re-naming bacteriophages is symptomatic for the confusing nomenclature of phages infecting many different organisms, and underlines the urgent need for a strong, reliable and unambiguous classification scheme which should be based primarily on molecular data. Most bacteriophage names do not provide immediate recognition of host and origin and often have dual meanings (e.g. TP can stand for ‘transducing phage’ or ‘B. *thuringiensis* phage’). Recently, a rational scheme for phage nomenclature has been proposed by Kropinski *et al.* [26], which would resolve ambiguities by a name prefix composed of virus type identifier, host abbreviation, virus family and specific designation. However, the TP21 phages represent the rare case where even such a robust system alone would fail, since all TP21 phages are from the same virus type, family and host organism. In these cases, the addition of a suffix, as proposed here, provides means of resolution of the naming disorder, even it if results in re-naming of the phages with unpredictable side-effects to citation of scientific literature.

## Figures and Tables

**Table 1.** General features, database matches and functional assignments of coding sequences (cds) of the *Bacillus* phage TP21-L genome for which homologies (e-value < 0.01) to known proteins could be found. Molecular mass (MM) and isoelectric points (pI) of the gene products (gp) are indicated. An asterisk indicates transcription on the complementary strand.

**cds**	**start**	**stop**	**MM (kDa)**	**pI**	**amino acid homologies (best hits) and deduced putative function**
1	10	489	17.8	6.6	Phage terminase small subunit (various *Streptococcus*, *Bacillus, Clostridium* and *Enterococcus* prophages*)*
2	808	2127	51.8	8.4	Putative large terminase subunit (*Staphylococcus* phage CNPH82)
3	2299	3771	56.8	5.0	SPP1 family phage portal protein; ORF003 Staphylococcus phage 187
4	3775	4800	39.9	9.4	Putative head protein (*Clostridium* phage phi CD119); SPP1 family phage head morphogenesis protein
7	5798	6634	30.8	5.9	Putative major head protein (various *Clostridium* prophages)
9	7040	7372	12.4	9.0	Putative phage head-tail adaptor (*Bacillus* prophages)
10	7372	7788	15.4	10.7	Phage protein, HK97 gp10 family (*Bacillus cereus* NVH0597-99 and others)
15	9651	12494	99.9	5.3	Phage tail tape measure protein, TP901 (*Bacillus* prophages)
16	12506	14020	57.8	6.0	Phage putative tail component (*Bacillus cereus* subsp. cytotoxis NVH 391–98); phage tail fiber protein (Bacillus phage Gamma)
17	14024	19144	192.6	6.3	Phage minor structural protein (*Bacillus* prophages)
19	19500	20291	29.7	8.9	Lysin (*Bacillus* phage IEBH); prophage LambdaBa01, N-acetylmuramoyl-L-alanine amidase
20*	20372	21754	53.7	8.9	Recombinase (*Bacillus* and *Paenibacillus* prophages)
21*	21754	22386	23.9	8.9	Transcriptional regulator (*Bacillus thuringiensis* serovar monterrey BGSC 4AJ1)
22	22645	22893	9.8	9.4	Cro-like protein, phage associated (*Lactobacillus* phage Sal2)
32	26585	27400	32.5	8.9	ORF016 (*Staphylococcus* phage 42E); primosome, DnaD subunit (*Geobacillus* sp.)
34	27570	28313	29.2	9.2	Rha family regulatory protein (*Bacillus weihenstephanensis* KBAB4); phage regulatory protein, Rha family (*Paenibacillus* sp. JDR-2)
40	30404	31744	51.3	6.1	Replicative DNA helicase (*Bacillus thuringiensis* serovar tochigiensis BGSC 4Y1)
